# Supplementary material for: Chemical mimicry of viral capsid self-assembly via corannulene-based pentatopic tectons
Source: Nat Commun. 2019 Aug 1;10:3443. doi: 10.1038/s41467-019-11457-6 (PMC6671967; doi:10.1038/s41467-019-11457-6)
Supplement: Supplementary file 1 — Supplementary Information [file 41467_2019_11457_MOESM1_ESM.pdf]

Supplementary Information

Chemical mimicry of viral capsid self-assembly via corannulene-based pentatopic tectons

Chen et al.

Supplementary Methods

Materials and general methods. Unless otherwise noted, reagents and solvents were used as received from Fisher Scientific and Sigma-Aldrich without further purification. Column chromatography was conducted using silica gel (75-200 μm) and amino-functionalized silica gel (75-200 μm) from Fuji Silysia GS series, and basic Al_2O_3 (50-200 μm) from Acros. ^{1}H and ^{13}C NMR spectra were recorded at 25 °C on a Varian Mercury NMR 400 spectrometer or a Bruker NMR 500 spectrometer, where chemical shifts (δ in ppm) were determined with respect to the nondeuterated solvents as a reference. The transmission electron microscope (TEM) micrographs were acquired on a Hitachi Model H-7650 microscope operating at 120 kV, and the high-resolution TEM micrographs were recorded on a JEOL JEM-2100F microscope operating at 200 or 120 kV. TEM samples were prepared by drop-casting a sample solution (10^{-6}~10^{-7} M) in acetonitrile onto a carbon-coated copper grid and then dried *in vacuo* for 24 h. Atomic force microscopy (AFM) was conducted on a Bruker Dimension Icon AFM system with ScanAsyst mode and the data were processed by NanoScope Analysis version 1.5 (Bruker Software, Inc.). AFM samples were prepared by spin-coating (3000 rpm for 30 s) a sample solution (10^{-6}~10^{-7} M) on a freshly cleaved mica surface.

High resolution TEM studies (Supplementary Figs 2-5) were conducted in a FEI Titan 80-300keV FEG S/TEM, at an accelerating voltage of 300 kV. We worked in both TEM mode with a Cs corrector and STEM mode with the HAADF (high-angle annular dark field) detector, and chemical analysis was done by an EDAX EDS detector and Gatan GIF EELS detector. Specimens for the TEM were prepared by dip-coating. A solution of 0.25 wt.% PS in chloroform was prepared, and 300 square mesh Cu TEM grids were dipped into the solution and dried at room temperature. Due to the surface energy of PS, the films form a concave shape between the Cu grid lines, resulting in thin regions near the center.

Mass spectrometry and ion mobility experiments. Mass spectrometry and traveling wave ion mobility (TWIM) experiments were conducted on a Waters Synapt HDMS G2 instrument with a LockSpray ESI source, using the following parameters: ESI capillary voltage, 3.0-4.5 kV; sample cone voltage, 20-50 V; extraction cone voltage, 0 V; desolvation gas flow, 800 L h^{-1} (N_2); trap collision energy (CE), 4 V; transfer CE, 0 V; trap gas flow, 2.0 mL min^{-1} (Ar); source temperature, 30 °C; and desolvation temperature, 30 °C. All samples were dissolved in MeCN and then infused into the ESI source at a flow rate of 6 μL min^{-1} by a syringe pump (KDS-100, KD Scientific). For TWIM experiments, the helium cell gas flow was held at 180.0 mL min^{-1} and the ion mobility cell gas flow was held at 90.0 mL min^{-1} (N_2). The TWIM DC

traveling wave velocity and height were set as 683 m s^{-1} and 26.3 V, respectively. Data were collected and analyzed by using MassLynx 4.1 and DriftScope 2.4 (Waters). Matrix-assisted laser desorption/ionization coupled with time-of-flight detector (MALDI-TOF) mass spectrometry was conducted on a Bruker autoflex spectrometer with a 355 nm frequency-tripled Nd:YAG SmartBean laser. 1.0 μL of α-cyano-4-hydroxycinnamic acid (CHCA) matrix solution (10 mg mL^{-1} in CH_3CN) was first deposited on a MALDI plate and air-dried. Aliquots of sample solution (1 mg mL^{-1} in $CHCl_3$) were then added onto the matrix spots for characterization.

Collision cross-section calibration. The calibration curve was established according to the protocol listed in the literature[1] using published collision cross-sections of polyalanine, cytochrome c (bovine), reserpine, lysozyme, and insulin (human). A plot of corrected drift times versus corrected cross-sections of calibrants fitted with power functions was used as a calibration curve for cross-section measurements.

Small-angle X-ray scattering. For SAXS measurements, all the SAXS profiles were acquired at the 23A beamline of the National Synchrotron Radiation Research Center (NSRRC) in Taiwan. The X-ray with a wavelength of λ = 0.827 Å (15 keV) and a typical current of 300 mA was used. A 2D PILATUS 1M-F detector was used to capture the scattering patterns. The sample-to-detector distance is 2469.15 mm to give a q range of 0.06 to 0.35 $Å^{-1}$. q is the scattering vector, related to the scattering angle (2θ) and the photon wavelength (λ) by $q = 4\pi\sin(\theta)/\lambda$. The 2D SAXS images were converted into 1D SAXS profiles $I(q)$ to q followed by background subtraction. Igor Pro. 6.3 software was used to analyze all SAXS data and the models were used for fitting the form-factor from the NCNR package. All sample solutions and solvents were filtered into empty vials through a 220 nm syringe filter before the measurement. The sample solution was sealed between two quartz windows during the measurement. The analytical solution was prepared at a concentration of 0.5 mg mL^{-1} at 25 °C. Acetonitrile was used as blank to conduct background subtraction.

Molecular modeling. Energy-minimized structures were obtained following the same settings in the literature[1]. Calculations were proceeded with Anneal and Geometry Optimization functions in Forcite module of Materials Studio version 6.1 program (Accelrys Software, Inc.). For each structure, 200 conformations after annealing were generated and converted to collision cross-sections using projection approximation (PA) and trajectory method (TM) in MOBCAL[2].

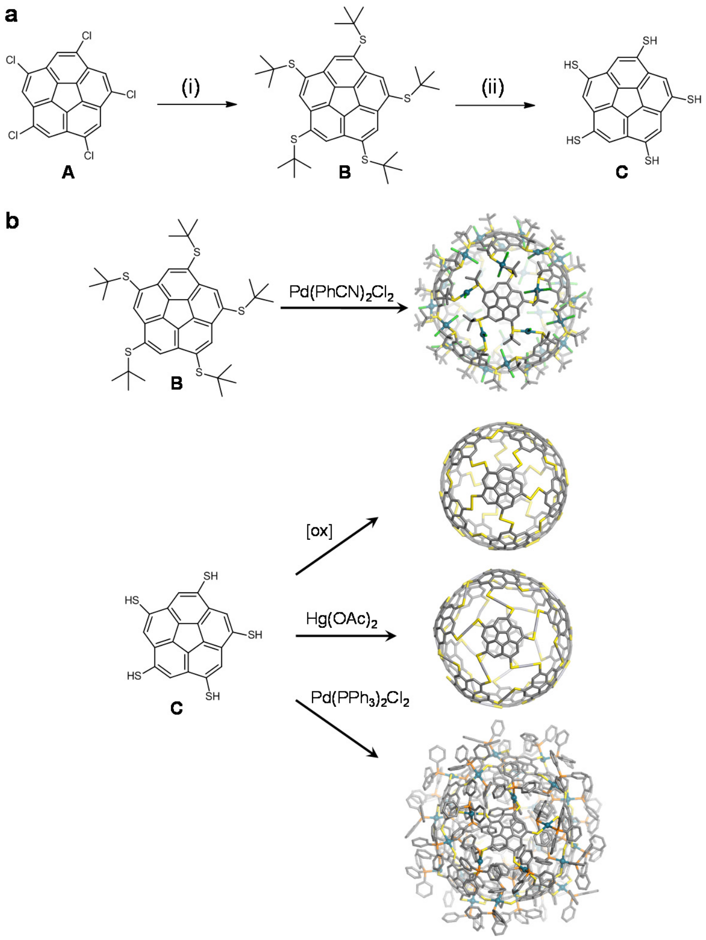

Supplementary Figure 1. a Reagents and conditions for the synthesis of the corannulene tectons **B** and **C**: (i) *t*-BuSNa, DMI, r.t.; (ii) TFA, HOTf, toluene, 80 °C. **b** Attempted assembly of sulfur-containing corannulene derivatives, **B** and **C**, to the modeled chemical capsids.

Corannulene tectons with sulfur-based ligands. Our initial efforts focused on 1,3,5,7,9-pentamercaptocorannulene, **C**, which could lead to the desired capsid via reversible formation of disulfide bonds. We prepared this compound from **A** via nucleophilic substitution with sodium 2-methyl-2-propanethiolate to afford 1,3,5,7,9-penta(*t*-butylsulfanyl)corannulene, **B**[3], followed by acid-catalyzed dealkylation of the thioethers to produce **C** (Supplementary Fig. 1a). Unfortunately, treatment of **C** with either diphenyl disulfide or Ellman's reagent in the presence of various bases and solvents resulted in precipitates of insoluble products.

Switching to a metal binding strategy (Supplementary Fig. 1b), we employed either Pd(II) or Hg(II) as linear linkers in order to interconnect the pentatopic sulfur ligands, **B** and **C**. Addition of $Pd(PPh_3)_2Cl_2$ and *n*-butyl amine to a solution of **C** in $CHCl_3$ resulted

in a homogenous orange-red mixture with no apparent formation of metallic palladium. In order to analyze this mixture by high-resolution transmission electron microscopy (TEM) we entrapped it within a solid polymeric matrix[4]. Thus, blending the reaction mixture with soluble polystyrene in $CHCl_3$ (0.25% w/w) and letting it dry at room temperature under atmospheric pressure on the microscope copper grid produced appropriate thin films for TEM analyses. The films contained spherical objects of the expected diameter (2-3 nm), which were darker than the background polymer and exhibited pentagonal symmetry (Supplementary Figs 2a-b).

Similar experiments were carried out with Hg(**II**). Thus, **B** was mixed with $Hg(OAc)_2$ and potassium *t*-butoxide in DMF and the mixture was kept at 70 ˚C for two days. TEM and STEM analyses of this mixture entrapped within films of polymerized tetraethyl orthosilicate (TEOS) showed images of spherical objects with diameters of 2-3 nm (Supplementary Fig. 3). Energy-dispersive X-ray spectroscopy (EDS) analysis of the blank film showed only the background matrix of silicon and oxygen whereas similar analyses of the spherical objects showed the expected presence of Hg, S and C (Supplementary Fig. 4).

As the penta-thioether, **B**, is much more soluble than **C**, we assumed that it could form a spherical capsid upon coordination to various metal centers. Furthermore, the solid-state structure of **B**[3], showed that all *t*-butyl groups prefer an *exo* conformation in which they occupy the exterior of the corannulene bowl, thus making the sulfur atoms available for convenient metal coordination at the *endo* (concave) face of the molecule. We mixed **B** with $Pd(PhCN)_2Cl_2$ and then entrapped the mixture within a polystyrene matrix. TEM analysis showed dark spheres of 2-3 nm diameter (Supplementary Fig. 2c). Aggregates of these objects were also analyzed by EDS, indicating the presence of palladium. Electron energy loss spectroscopy (EELS) analysis of those objects showed characteristic bands of Pd, S and C (Supplementary Fig. 5). Unfortunately, these reaction products were insufficiently soluble to allow for either NMR or MS analyses.

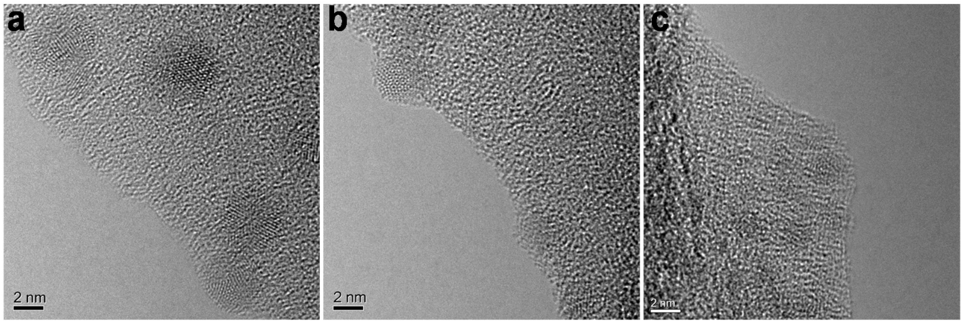

Supplementary Figure 2. TEM analysis of the crude reaction mixtures entrapped within a polystyrene matrix. **a**, **b** Pd(**II**)-mediated assembly of **C**. **c** Pd(**II**)-mediated assembly of **B**.

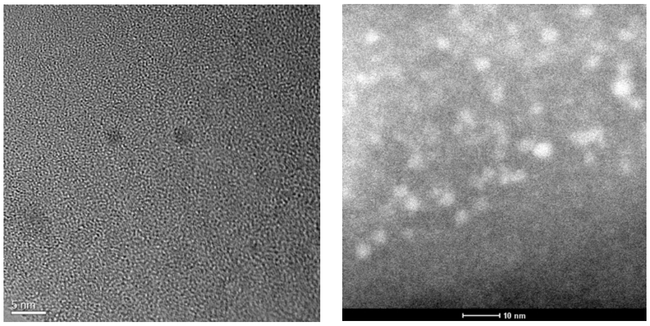

Supplementary Figure 3. TEM (left) and STEM (right) images of the Hg(**II**)-mediated assembly of **C**.

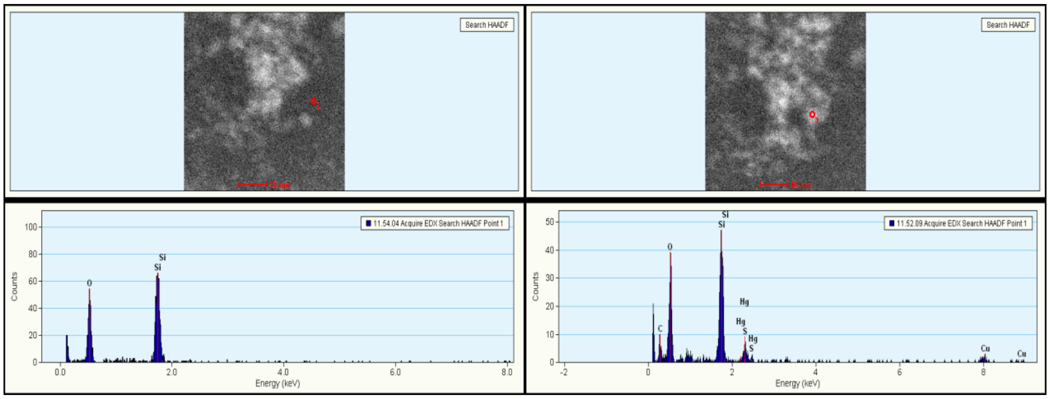

Supplementary Figure 4. STEM images of the Hg(**II**)-mediated assembly of **C** along with chemical analysis by EDS of a spot on the film (left) and on the aggregate (right).

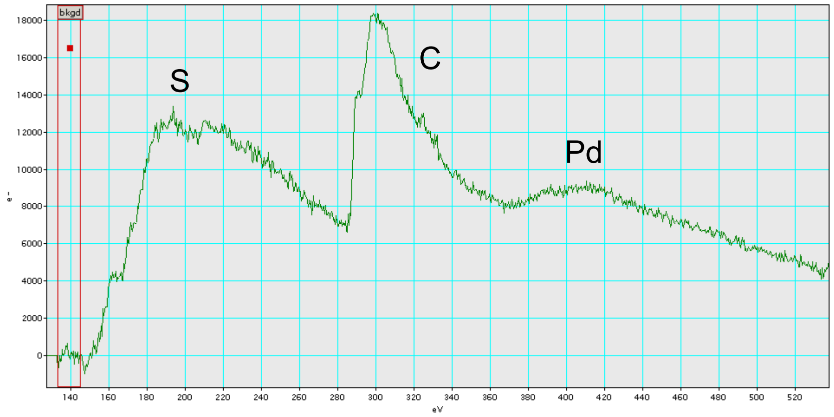

Supplementary Figure 5. EELS spectrum of a single point in the film shown in Supplementary Fig. 2c.

Synthesis of building blocks 1 and 2. 1,3,5,7,9-Pentamethoxy-2,4,6,8,10-pentabromocorannulene, **1**, was prepared by the Cu(I)-catalyzed Ullmann condensation reaction between methanol and sym-pentachlorocorannulene, and the subsequent electrophilic bromination, as described by Pogoreltsev et al[5]. 4-(4′-2,2′:6′,2″-Terpyridyl)phenylboronic acid, **2a** (R = H), and 4-(4′-4,4″-di-*t*-butyl-2,2′:6′,2″-terpyridyl)phenylboronic acid, **2b** (R = *t*-butyl), were synthesized according to the reported procedure of Wang et al[6].

Synthesis of pentagonal ligand 3a (Fig. 2a). To a degassed two-neck flask containing compound **1** (100.0 mg, 125.0 μmol), **2a** (444.0 mg, 1.3 mmol), and Na_2CO_3 (134.0 mg, 1.3 mmol), a mixed solvent (46 mL) of toluene/H_2O/*t*-BuOH (3:3:1, v/v/v) was added. After being purged with N_2 for 30 min, $Pd(PPh_3)_4$ (72.0 mg, 63.0 μmol) was added into the mixture, which was then refluxed for 2 days under N_2 atmosphere. After cooling to r.t., the mixture was extracted with $CHCl_3$ and washed with saturated $NH_4Cl_{(aq)}$. The combined organic layer was dried over $MgSO_4$ and then evaporated to dryness under reduced pressure. The residue was subjected to flash column chromatography (basic Al_2O_3, $CHCl_3$), and then further separated by another column chromatography (amino-functionalized SiO_2, CH_2Cl_2/hexane) to give **3a** as a yellow solid (120.0 mg, 62.0 μmol) in 49% yield. ^{1}H NMR (400 MHz, $CDCl_3$): δ (ppm) 8.79 (s, 10H), 8.69 (d, J = 4.0 Hz, 10H), 8.64 (d, J = 8.0 Hz, 10H), 7.97 (d, J = 8.3 Hz, 10H), 7.83 (td, J = 7.7 Hz, 1.8 Hz, 10H), 7.76 (d, J = 8.2 Hz, 10H), 7.30 (ddd, J = 7.6 Hz, 4.8 Hz, 1.2 Hz, 10H), and 2.91 (s, 15H). ^{13}C NMR (100 MHz, $CDCl_3$): δ (ppm) 156.51, 156.23, 155.80, 150.28, 149.04, 138.15, 136.86, 136.71, 131.68, 131.44, 131.07, 126.18, 125.63, 123.65, 121.23, 118.91, and 61.53. MALDI–TOF MS (*m/z*): 1936.6808 $[M]^+$ (calcd *m/z* = 1936.6892).

Synthesis of pentagonal ligand 3b (Fig. 2a). By the same protocol as that for **3a**, **3b** was obtained in 57% yield (180.0 mg, 72.0 μmol) from **1** (100.0 mg, 125.0 μmol), **2b** (437.0 mg, 0.9 mmol), Na_2CO_3 (100.0 mg, 0.9 mmol), and $Pd(PPh_3)_4$ (72.0 mg, 63.0 μmol). ^1H NMR (400 MHz, $CDCl_3$): δ (ppm) 8.80–8.73 (m, 20H), 8.60 (d, J = 5.3 Hz, 10H), 7.96 (d, J = 8.3 Hz, 10H), 7.74 (d, J = 8.2 Hz, 10H), 7.31 (dd, J = 5.3 Hz, 2.0 Hz, 10H), 2.91 (s, 15H), and 1.41 (s, 90H). ^{13}C NMR (100 MHz, $CDCl_3$): δ (ppm) 160.57, 156.54, 156.17, 155.87, 150.42, 149.01, 138.10, 136.97, 131.66, 131.42, 131.12, 126.24, 125.68, 120.94, 118.74, 118.14, 61.55, 34.91, and 30.50. MALDI–TOF MS (m/z): 2497.3118 $[M]^+$ (calcd m/z = 2497.3152).

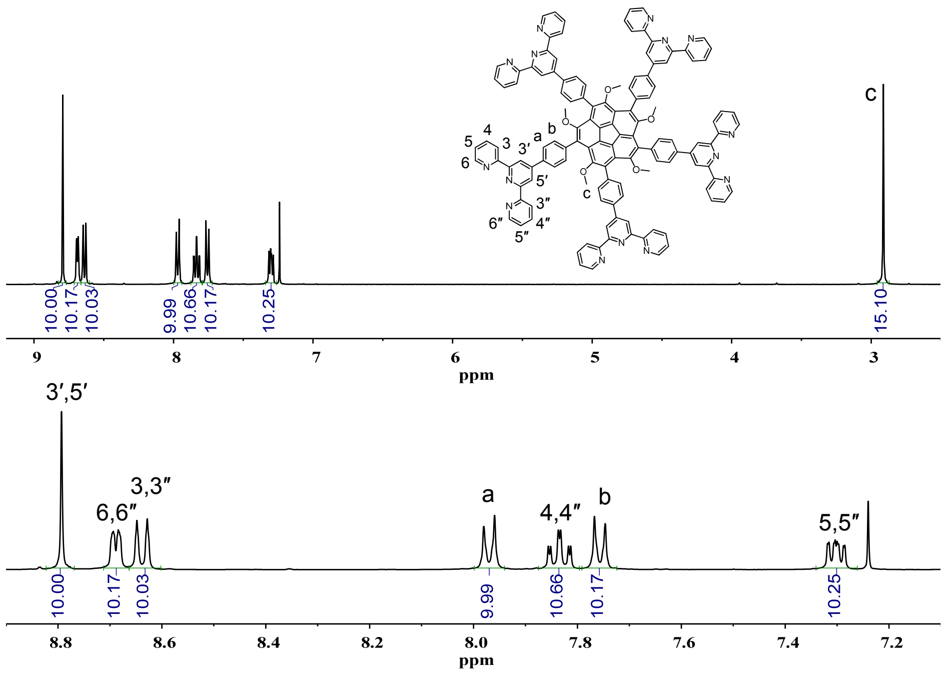

Supplementary Figure 6. ^1H NMR spectrum of ligand **3a**.

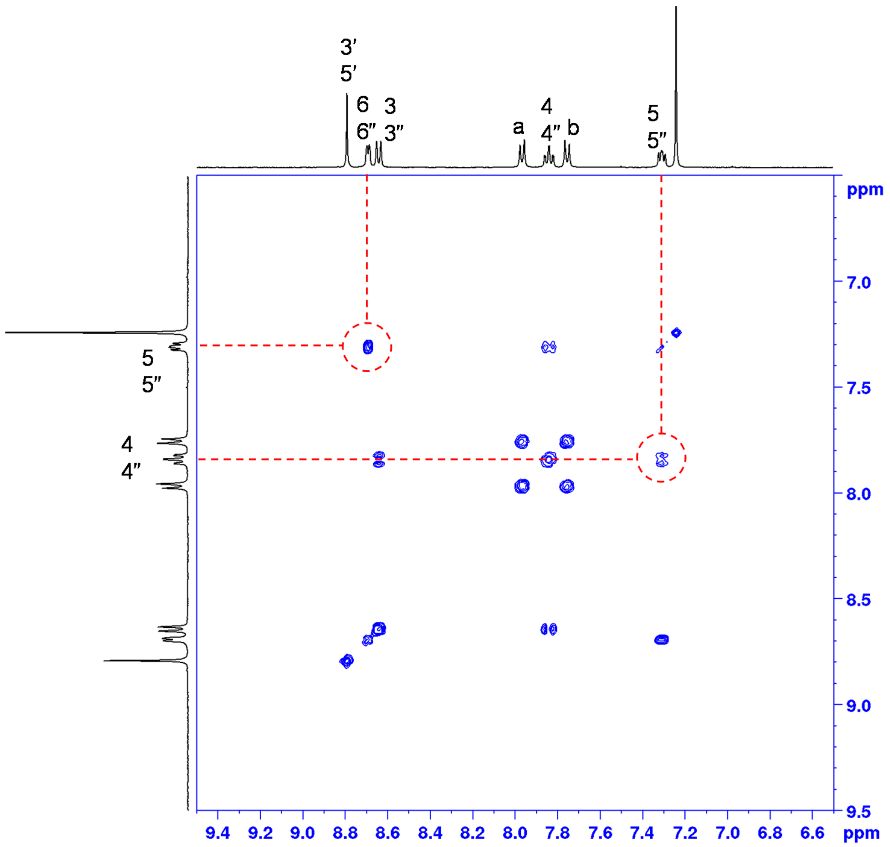

Supplementary Figure 7. COSY spectrum of ligand **3a**.

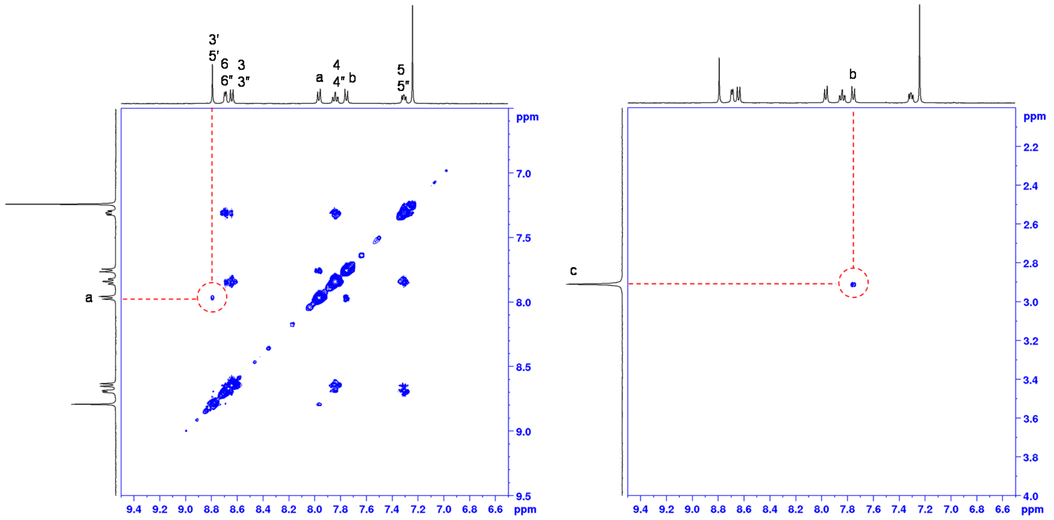

Supplementary Figure 8. NOESY spectrum of ligand **3a**.

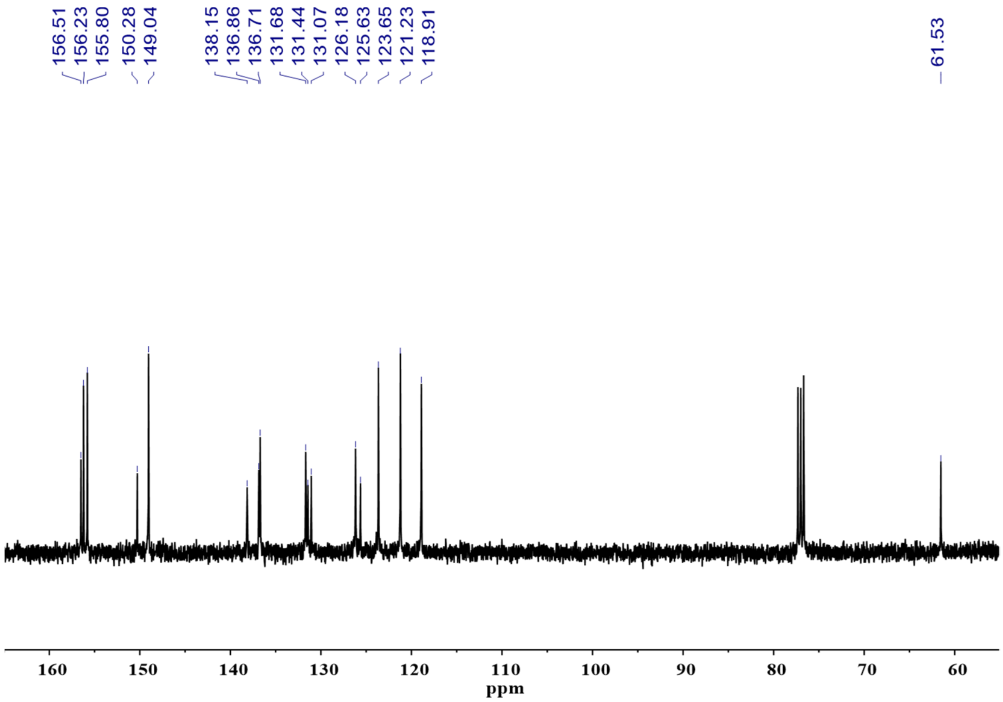

Supplementary Figure 9. ^{13}C NMR spectrum of ligand **3a**.

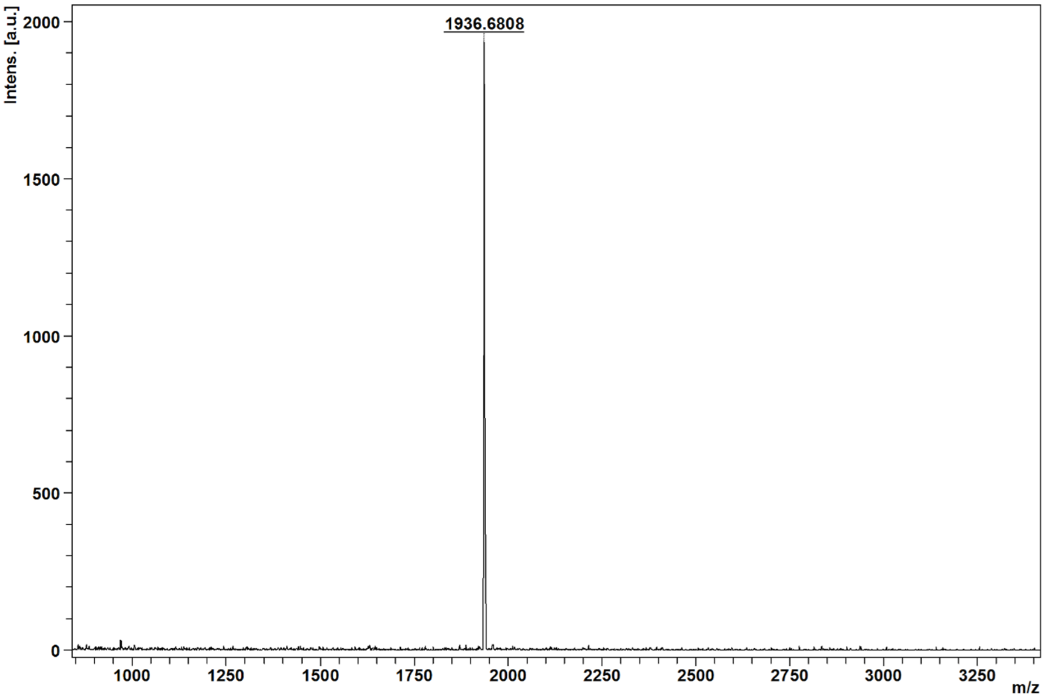

Supplementary Figure 10. MALDI-TOF-MS spectrum of ligand **3a**.

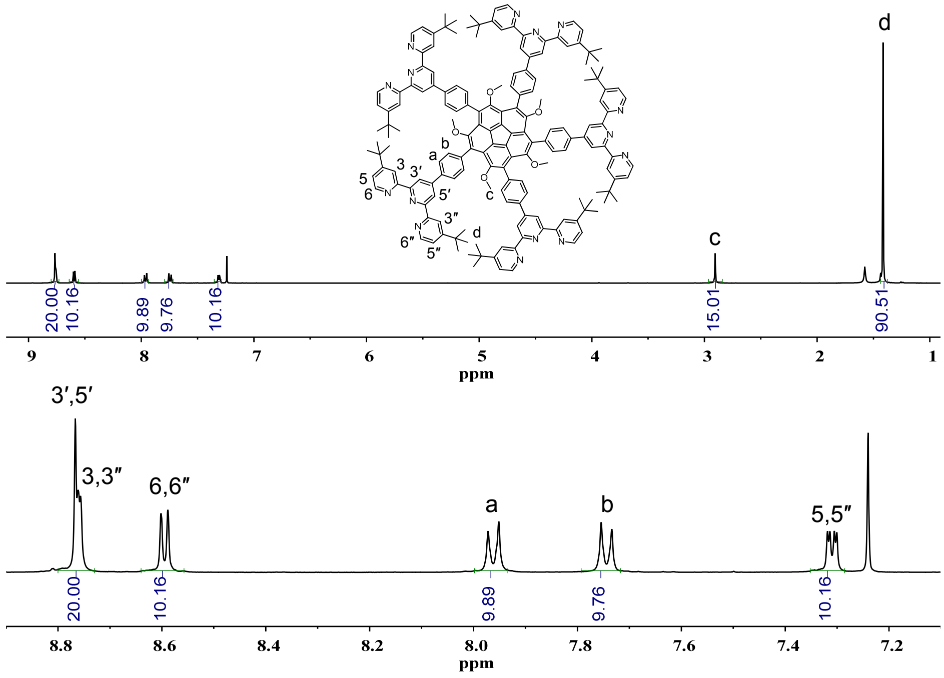

Supplementary Figure 11. ^{1}H NMR spectrum of ligand **3b**.

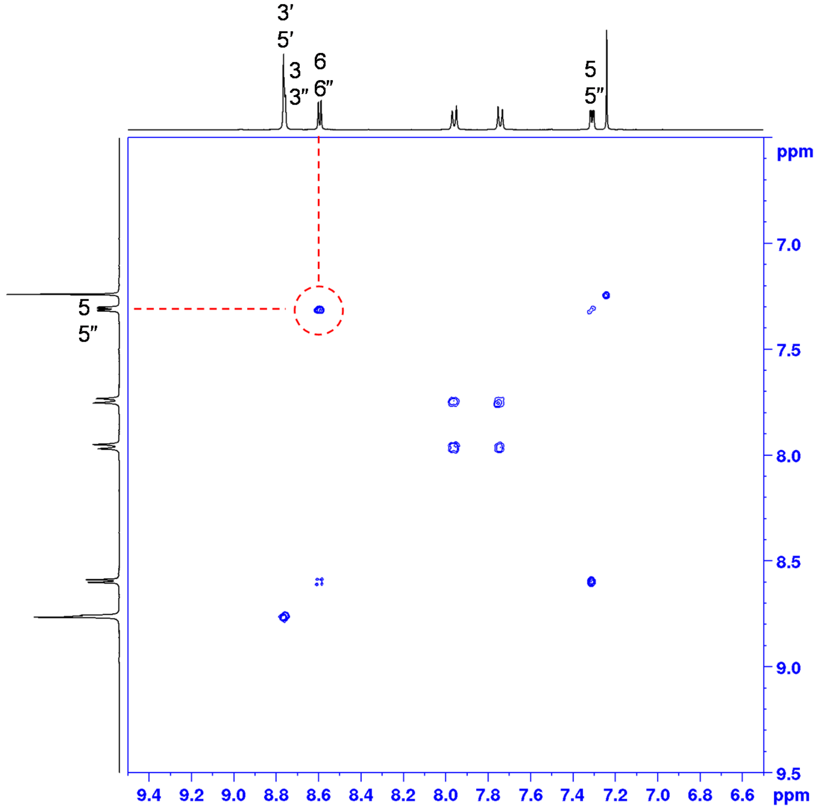

Supplementary Figure 12. COSY spectrum of ligand **3b**.

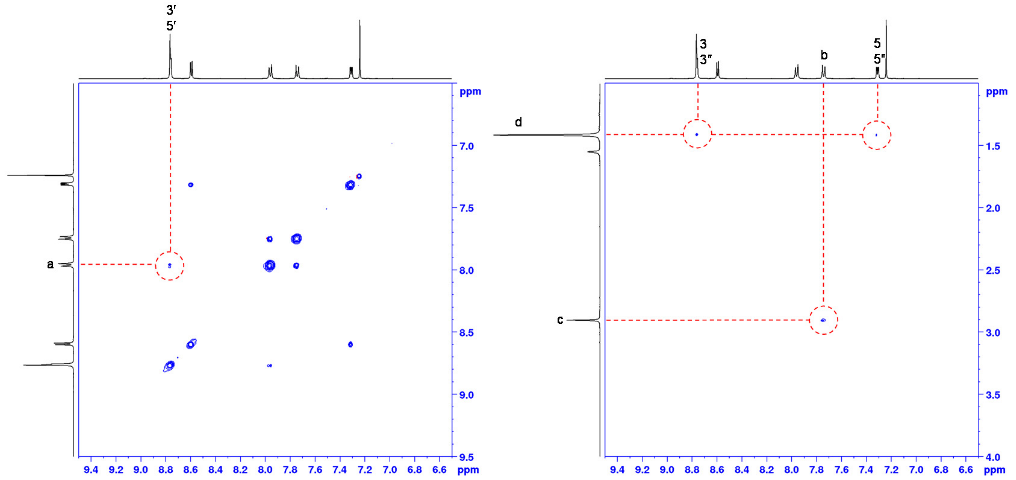

Supplementary Figure 13. NOESY spectrum of ligand **3b**.

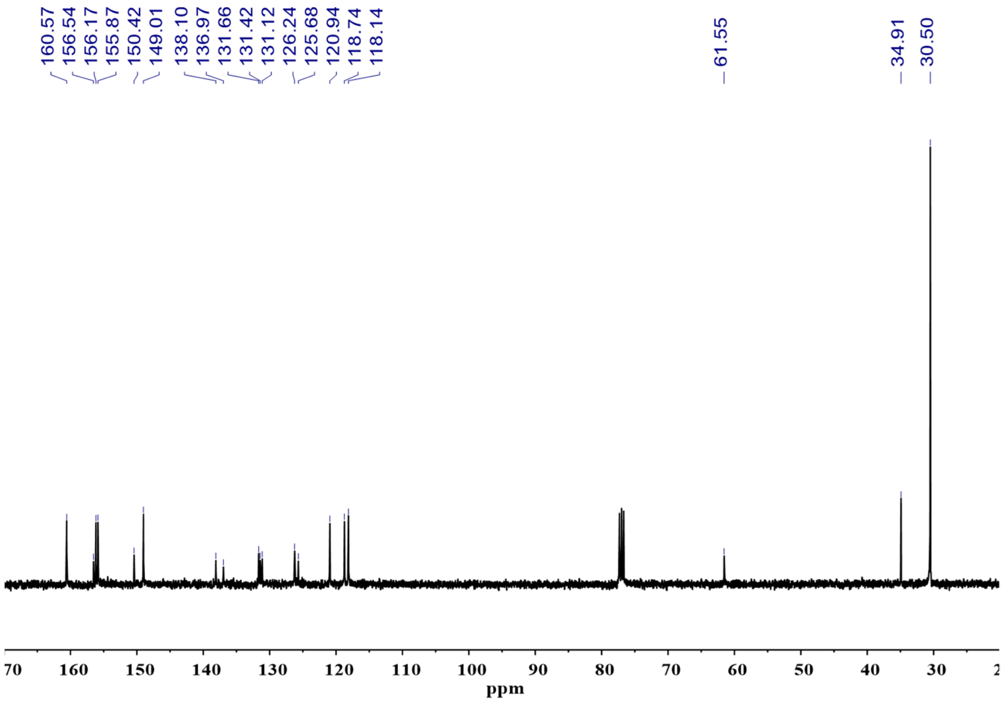

Supplementary Figure 14. ^{13}C NMR spectrum of ligand **3b**.

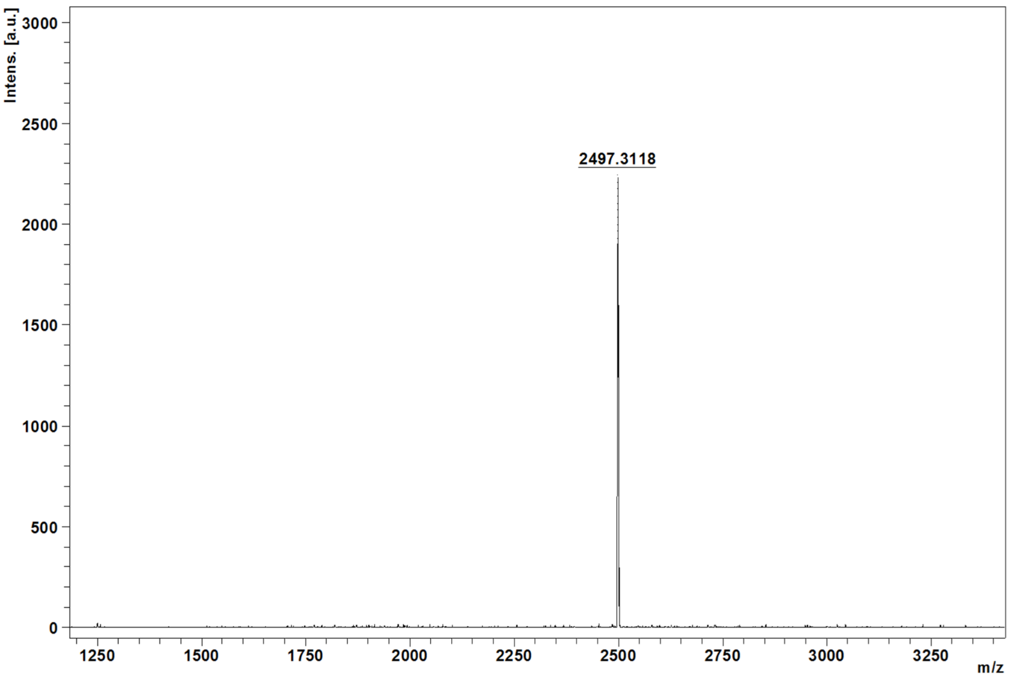

Supplementary Figure 15. MALDI-TOF-MS spectrum of ligand **3b**.

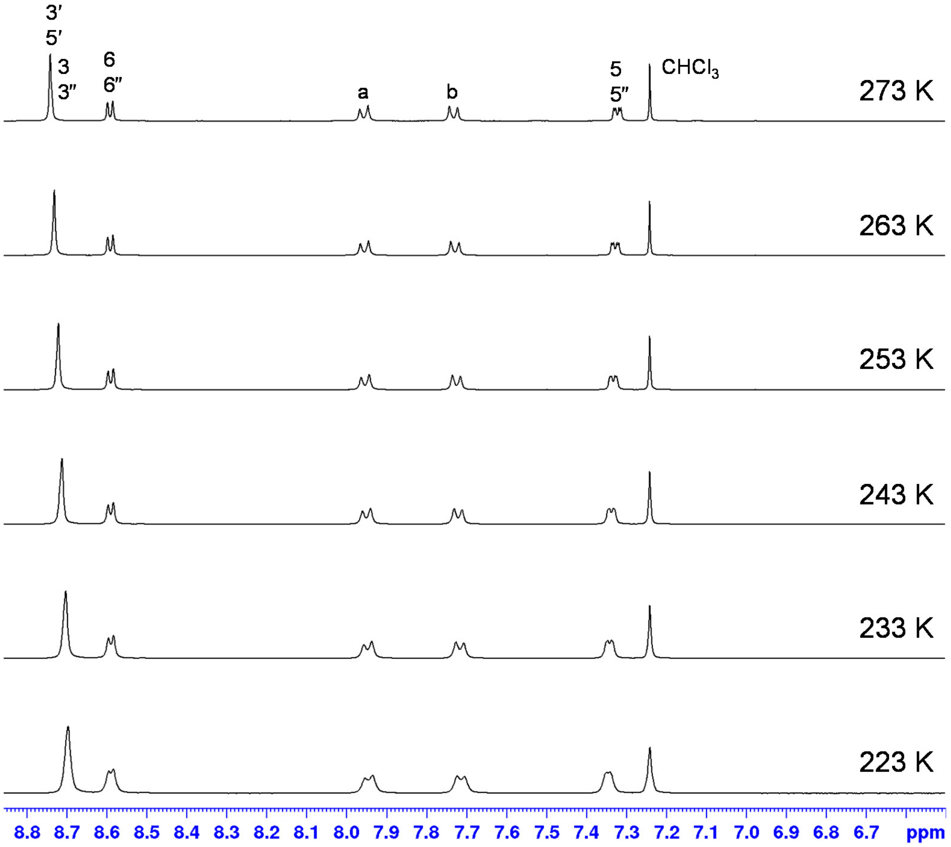

Supplementary Figure 16. Variable-temperature ^{1}H NMR spectra of **3b** taken in $CDCl_3$. No peak splitting for protons a an b is observed.

Synthesis of complex 4b. To a stirred $CHCl_3$ solution (5 mL) of **3b** (15.1 mg, 6.0 μmol), a MeOH solution (5 mL) of $Cd(NO_3)_2 \cdot 4H_2O$ (4.7 mg, 15.1 μmol) was added. The mixture was stirred at room temperature for 30 min, excess NH_4PF_6 (121.0 mg, 0.7 mmol) was added. The precipitate was filtered and washed with MeOH and H_2O, and then dissolved in MeCN (45 mL). The mixture was refluxed for 12 h, and then cooled to room temperature. The solvent was removed and the residue was washed with H_2O and MeOH, and then dried *in vacuo* to give complex **4b** (18.4 mg, 0.4 μmol) as a yellow powder in 88% yield. ^{1}H NMR (400 MHz, CD_3CN): δ (ppm) 9.02 (br, 120H), 8.64 (br, 120H), 8.43 (br, 60H), 8.25 (br, 60H), 8.13 (br, 60H), 7.91 (br, 120H), 7.61 (br, 60H), 7.42 (br, 120H), 3.24 (br, 180H), and 1.31 (br, 1080H). ^{13}C NMR (125 MHz, CD_3CN): δ (ppm) 167.04, 157.90, 156.15, 151.39, 150.23, 149.91, 141.40, 136.36, 133.15, 132.88, 132.49, 132.15, 128.49, 128.00, 127.31, 125.20, 122.91, 122.02, 63.33, 36.59, and 30.54. ^{113}Cd NMR (111 MHz, CD_3CN): δ (ppm) 275.64. ESI-MS (*m/z*): 2858.5088 $[M - 14PF_6]^{14+}$ (calcd *m/z* = 2858.5200), 2658.1443 $[M - 15PF_6]^{15+}$ (calcd *m/z* = 2658.1509), 2482.9465 $[M - 16PF_6]^{16+}$ (calcd *m/z* = 2482.9585), 2328.4741 $[M - 17PF_6]^{17+}$ (calcd *m/z* = 2328.4902), 2191.1046 $[M - 18PF_6]^{18+}$ (calcd *m/z* = 2191.1032), 2067.9722 $[M - 19PF_6]^{19+}$ (calcd *m/z* = 2067.9731), 1957.4626 $[M - 20PF_6]^{20+}$ (calcd *m/z* = 1957.4756), 1857.3510 $[M - 21PF_6]^{21+}$ (calcd *m/z* = 1857.3612), 1766.2952 $[M - 22PF_6]^{22+}$ (calcd *m/z* = 1766.2957), 1683.2117 $[M - 23PF_6]^{23+}$ (calcd *m/z* = 1683.2018), 1607.1583 $[M - 24PF_6]^{24+}$ (calcd *m/z* = 1607.1532), 1536.8730 $[M - 25PF_6]^{25+}$ (calcd *m/z* = 1536.8672), and 1472.3856 $[M - 26PF_6]^{26+}$ (calcd *m/z* = 1472.3748).

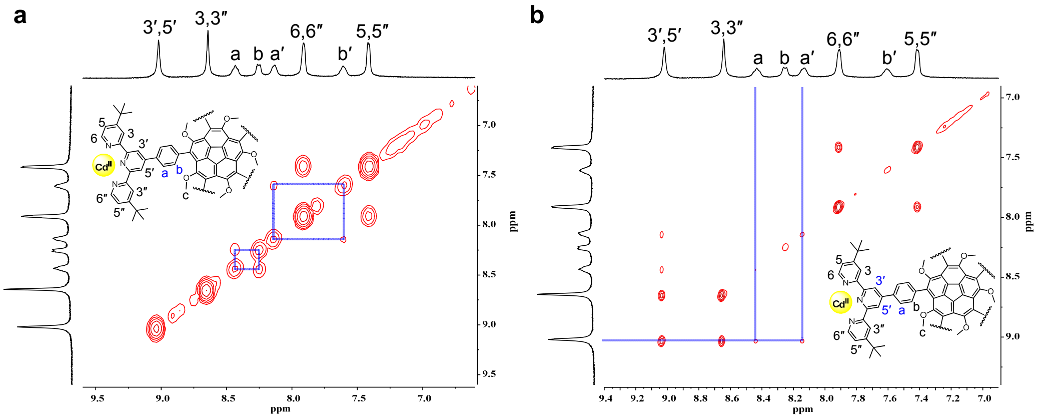

Supplementary Figure 17. Partial COSY (**a**) and ROESY (**b**) NMR spectra of **4b** in CD_3CN.

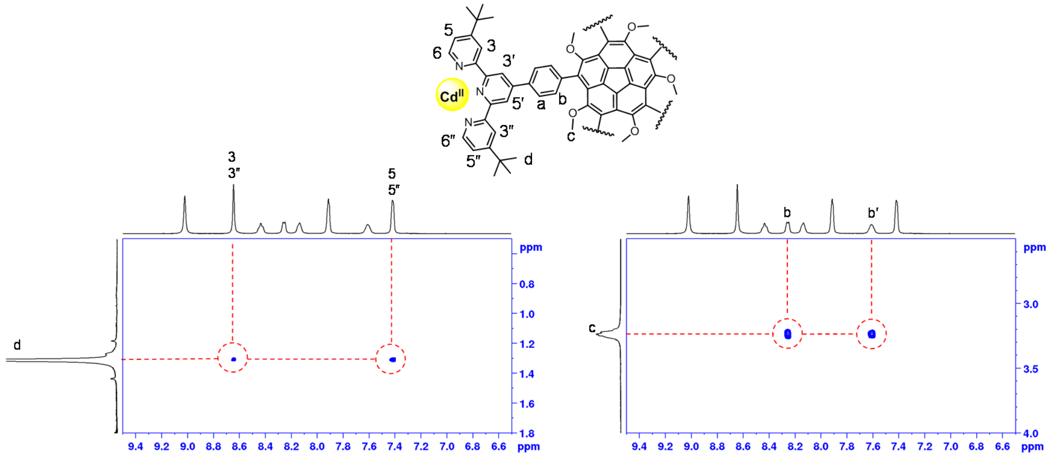

Supplementary Figure 18. Partial NOESY NMR spectra of **4b** in CD_3CN.

Slow rotation of the phenylene ring in the capsid. The ^{1}H-^{1}H 2D EXSY spectrum (Supplementary Fig. 19) revealed the exchange cross peaks (red) between the two signals for both a,a′ and b,b′, strongly supporting the two protons are involved in a slow exchange. This observation also excluded the possibility that the peak splitting is derived from the formation of diastereomers. Moreover, the exchange rate constant can be determined by Supplementary Equations 1 and 2 where k is the exchange rate constant and T_m is the mixing time used in the EXSY measurement[7]. Using signals b and b′ as an example, I_{bb} and $I_{b'b'}$ are the volumes of diagonal peaks and $I_{bb'}$ and $I_{b'b}$ are those of cross peaks. With varying mixing times, the exchange rate constant was estimated to be 3.64 s^{-1} at 25 °C.

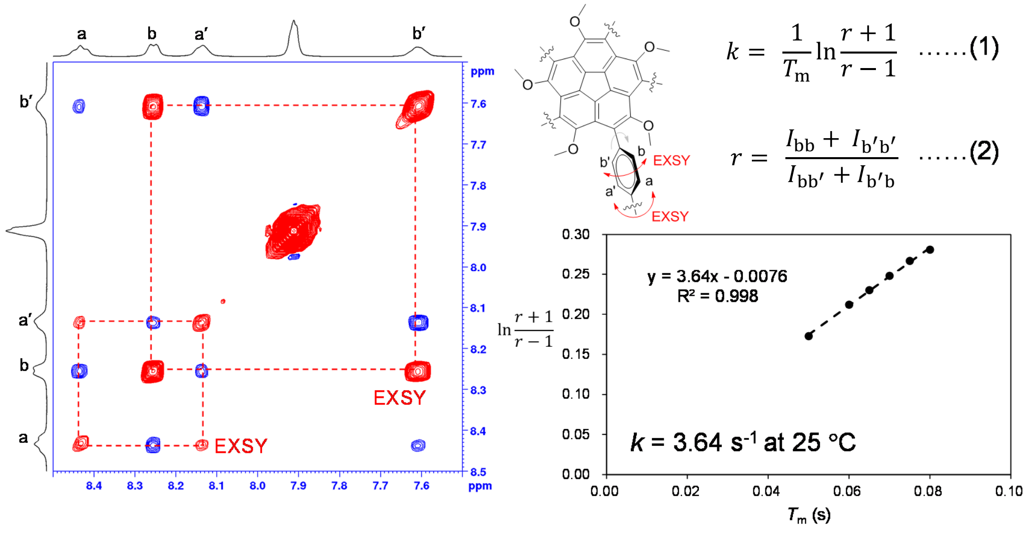

$$k = \frac{1}{T_m}\ln\frac{r+1}{r-1} \quad \text{……(1)}$$

$$r = \frac{I_{bb} + I_{b'b'}}{I_{bb'} + I_{b'b}} \quad \text{……(2)}$$

Supplementary Figure 19. ^{1}H-^{1}H EXSY NMR spectrum of **4b** taken in CD_3CN at 25 °C. The plot for estimating the exchange rate constant based on Supplementary Equations 1 and 2.

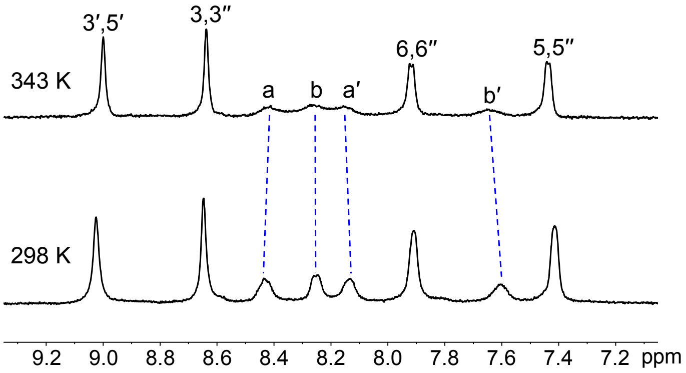

Supplementary Figure 20. Variable-temperature ^{1}H NMR spectra of **4b** at 343 (top) and 298 (bottom) K.

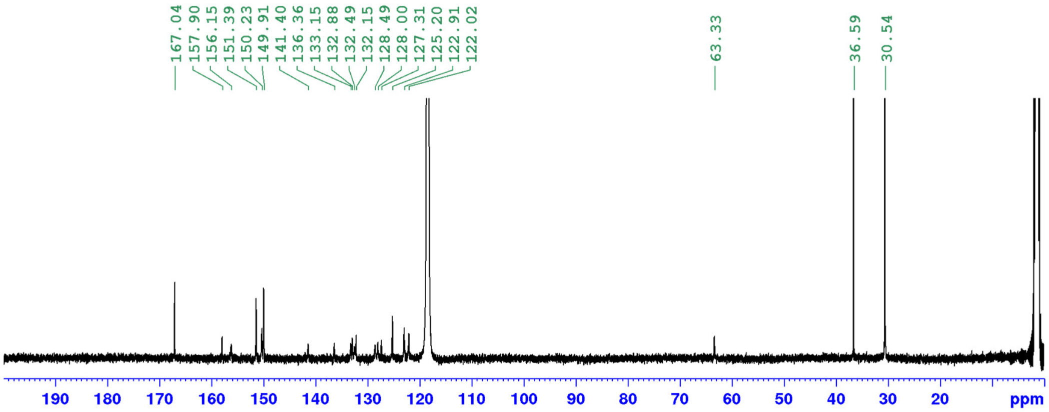

Supplementary Figure 21. ^{13}C NMR spectrum of **4b**.

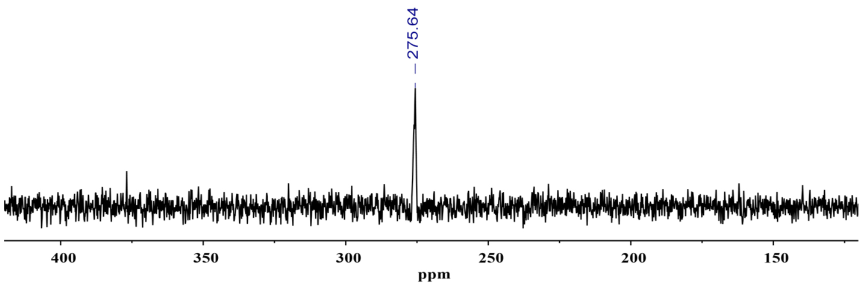

Supplementary Figure 22. ^{113}Cd NMR spectrum of **4b**.

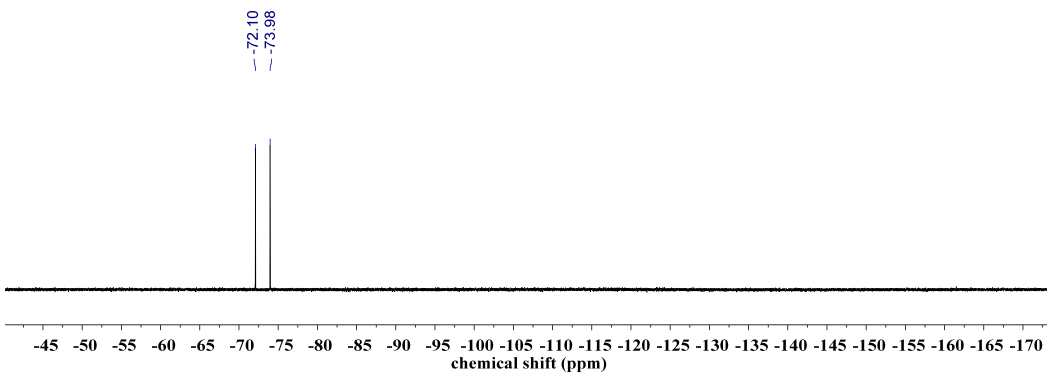

Supplementary Figure 23. ^{19}F NMR spectrum of **4b**.

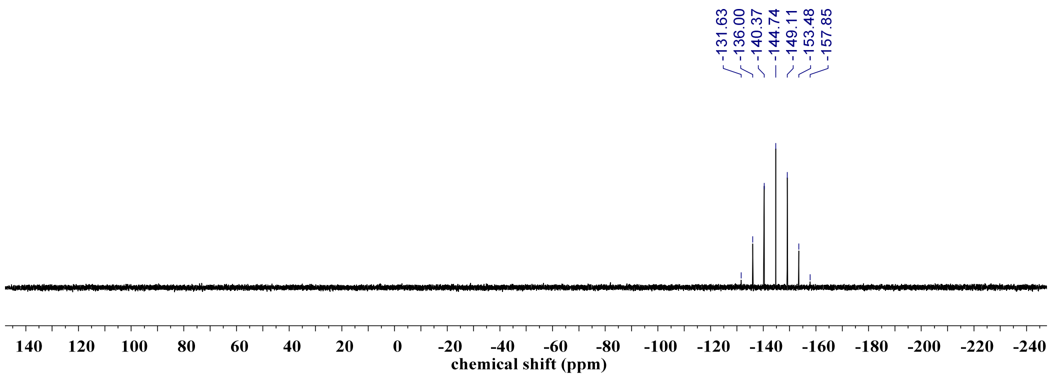

Supplementary Figure 24. ^{31}P NMR spectrum of **4b**.

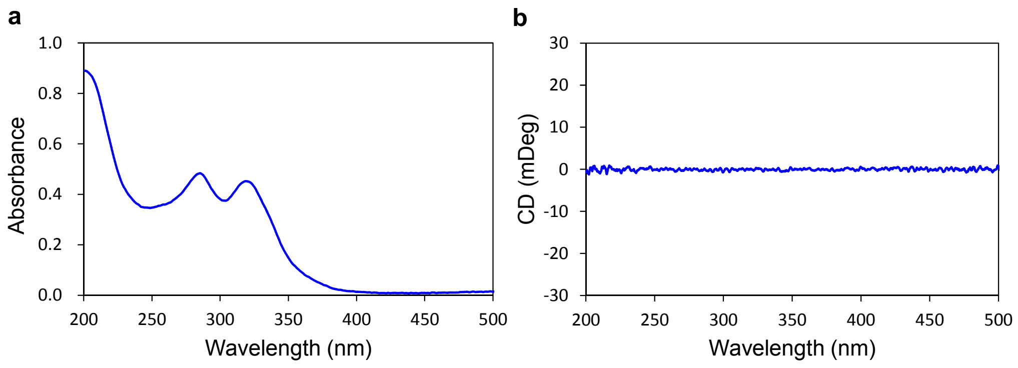

Supplementary Figure 25. UV-vis absorption (**a**) and CD (**b**) spectra of **4b** (1×10^{-5} M) in MeCN.

Resolution of two homochiral capsids by chiral HPLC. Chiral high-performance liquid chromatography (HPLC) was performed at 25 °C, using an analytical chiral packed column, Chiralpak AD-H (250 × 4.6 mm^2, 5 μm; Daicel Chemicals, Tokyo, Japan), on an Agilent Technologies 1200 Series HPLC system equipped with a G1315D DAD diode array detector. HPLC grade MeCN was used as an eluent with a flow rate at 0.6 mL/min and the measurements were monitored by a UV detector at λ = 290 nm.

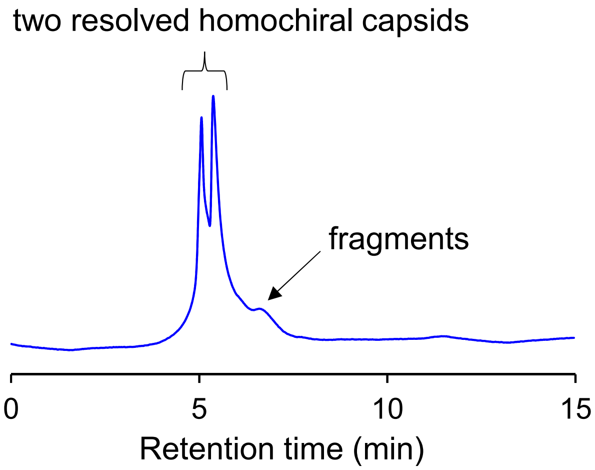

Supplementary Figure 26. Chiral HPLC chromatogram at 290 nm of **4b**. The fragments may be generated during separation due to disruption of labile coordination bonds.

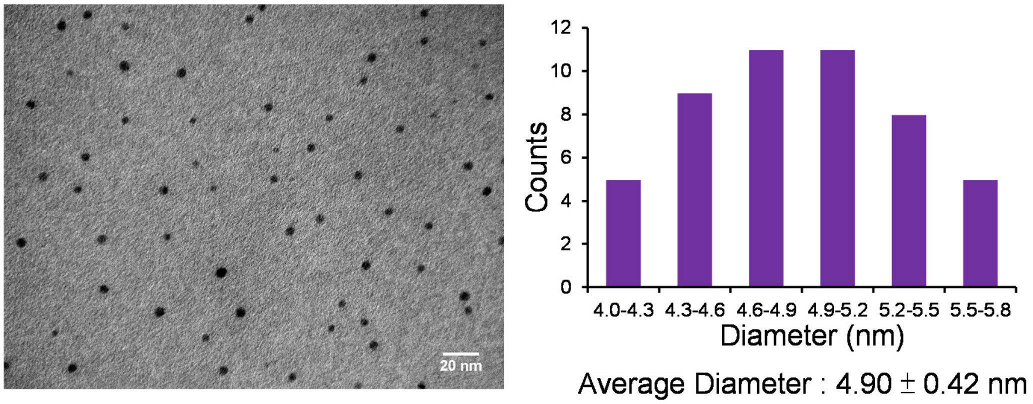

Supplementary Figure 27. TEM micrograph and statistical size distribution of **4b**.

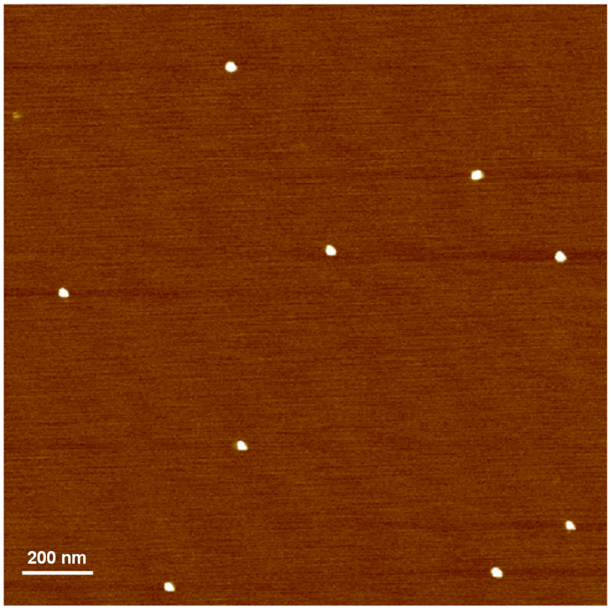

No.	Height (nm)	Diameter (nm)
1	6.73	42.65
2	5.98	39.32
3	6.21	39.55
4	5.86	45.98
5	5.66	41.94
6	5.34	47.39
7	5.34	40.98
8	5.88	39.79
9	5.23	35.02

Average Height: 5.80 ± 0.48 nm

Average Diameter: 41.40 ± 3.71 nm

Supplementary Figure 28. AFM image and statistical size distribution of **4b**. The tip-sample convolution effect[8] results in the low accuracy of the measured diameter.

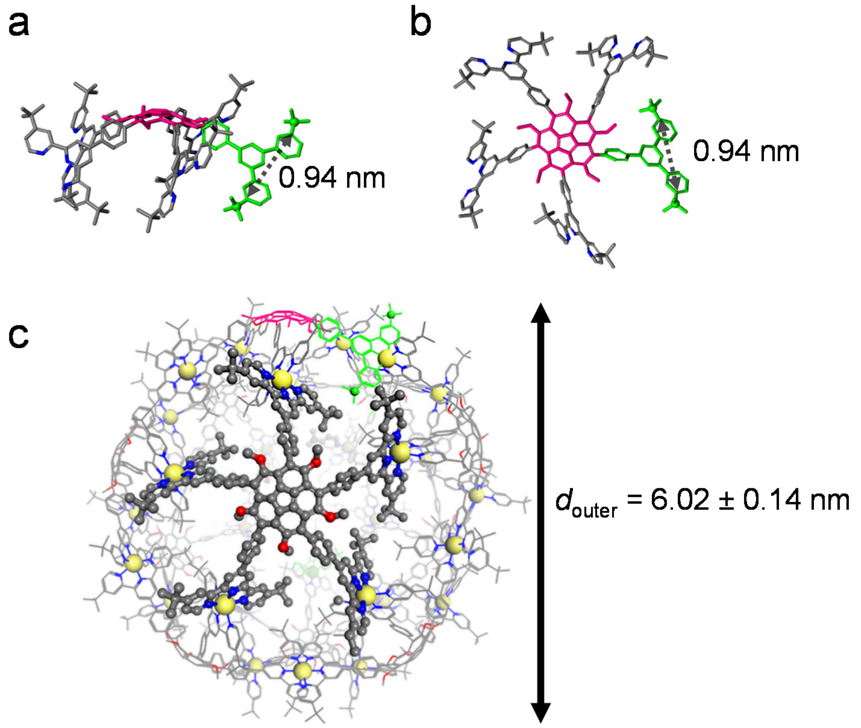

Supplementary Figure 29. Top (**a**) and side (**b**) views of the geometry-optimized structure of ligand **3b**. **c** Energy-minimized structure of **4b**, $[Cd_{30}L_{12}]$.

Supplementary Table 1. Experimental and theoretical collision cross-sections (CCSs) of **4b**.

Experimental

Charge State	*m/z*	Drift Time (ms)	CCS (Å^2)
25	1536.7188	6.5	3260.3
24	1607.0355	6.95	3281.8
23	1683.2843	7.28	3248.8
22	1766.2584	7.83	3268.5
21	1857.1813	8.38	3268.5
20	1957.0176	9.15	3303.8
21	1857.1813	7.72	3089.1
20	1957.0176	8.27	3084.3
19	2068.032	9.04	3112.6
18	2191.1292	9.81	3114.9
17	2328.2209	10.69	3114.3
16	2483.1863	11.91	3146.4
15	2658.2795	13.34	3174.8
Average			3189.8
Standard Deviation			83.1

Theoretical

Method	PA	TM
CCS (Å^2)	2872±11.6	3497±32.9

Supplementary Table 2. Radii (nm) of capsid **4b** derived from various methods.

r_{inner} (calcd)[a]	r_{outer} (calcd)[a]	r_{mean} (calcd)[a]	r_{inner} (SAXS)[b]	r_{outer} (SAXS)[b]	r_{mean} (SAXS)[b]	r_H (DOSY)[c]	r_{TEM} (TEM)[d]	r_{AFM} (AFM)[e]
**1.83 ± 0.07**	**3.01 ± 0.07**	**2.42 ± 0.05**	**1.97 ± 0.03**	**2.94 ± 0.05**	**2.46 ± 0.03**	**2.48 ± 0.05**	**2.45 ± 0.21**	**2.90 ± 0.24**

[a]Radii r_{inner} and r_{outer} calculated from the energy-minimized structure; $r_{mean} = (r_{inner} + r_{outer})/2$. [b]Radii calculated from the SAXS results; $r_{outer} = r_{inner}$ + shell thickness (t); $r_{mean} = (r_{inner} + r_{outer})/2$. [c]Hydrodynamic radius calculated by the Stokes-Einstein equation $D = k_BT/6\pi\eta r_H$ (k_B, Boltzmann constant; T = 298 K; η = 0.367 mPa·s, viscosity of CD_3CN at 298 K). [d]r_{TEM} is the average radius measured from the TEM micrographs. [e]r_{AFM} is the half of average AFM height.

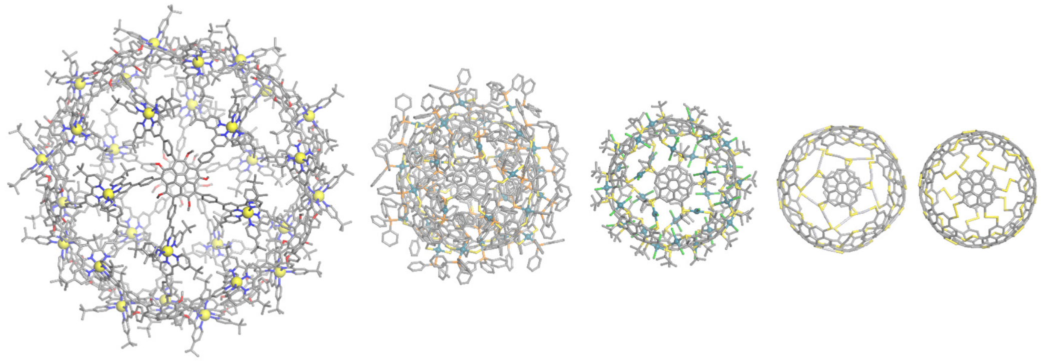

Supplementary Figure 30. Same-scale modeling of the various chemical capsids attempted in this study.

Supplementary References

1 Chan, Y.-T. *et al.* Design, synthesis, and traveling wave ion mobility mass spectrometry characterization of iron(II)– and ruthenium(II)–terpyridine metallomacrocycles. *J. Am. Chem. Soc.* **133**, 11967-11976 (2011).

2 Mesleh, M. F., Hunter, J. M., Shvartsburg, A. A., Schatz, G. C. & Jarrold, M. F. Structural information from ion mobility measurements: effects of the long-range potential. *J. Phys. Chem.* **100**, 16082-16086 (1996).

3 Angelova, P. *et al.* Chemisorbed monolayers of corannulene penta-thioethers on gold. *Langmuir* **29**, 2217-2223 (2013).

4 Thangadurai, P., Lumelsky, Y., Silverstein, M. S. & Kaplan, W. D. TEM specimen preparation of semiconductor–PMMA–metal interfaces. *Mater. Charact.* **59**, 1623-1629 (2008).

5 Pogoreltsev, A., Solel, E., Pappo, D. & Keinan, E. Deca-heterosubstituted corannulenes. *Chem. Commun.* **48**, 5425-5427 (2012).

6 Wang, C. *et al.* Self-assembly of giant supramolecular cubes with terpyridine ligands as vertices and metals on edges. *Chem. Sci.* **5**, 1221-1226 (2014).

7 Nikitin, K. & O'Gara, R. Mechanisms and Beyond: Elucidation of Fluxional Dynamics by Exchange NMR Spectroscopy. *Chem. Eur. J.* **25**, 4551-4589 (2019).

8 Radmacher, M., Fritz, M., Hansma, H. G. & Hansma, P. K. Direct observation of enzyme activity with the atomic force microscope. *Science* **265**, 1577-1579 (1994).
