# Supplementary material for: Chemical mimicry of viral capsid self-assembly via corannulene-based pentatopic tectons
Source: Nat Commun. 2019 Aug 1;10:3443. doi: 10.1038/s41467-019-11457-6 (PMC6671967; doi:10.1038/s41467-019-11457-6)
Supplement: Supplementary file 3 — Description of Additional Supplementary Files [file 41467_2019_11457_MOESM3_ESM.pdf]

Description of Additional Supplementary Files

File Name: Supplementary Movie 1

Description: Rotating molecular model of the geometry-optimized capsid **4b** (gray, carbon; blue, nitrogen; red, oxygen; yellow, cadmium). Hydrogen atoms and PF_6^- ions are omitted for clarity.
